# Rapid In-Field Volatile Sampling for Detection of *Botrytis cinerea* Infection in Wine Grapes

**DOI:** 10.3390/molecules28135227

**Published:** 2023-07-05

**Authors:** Liang Jiang, Morphy C. Dumlao, William A. Donald, Christopher C. Steel, Leigh M. Schmidtke

**Affiliations:** 1 Gulbali Institute, Charles Sturt University, Wagga Wagga, NSW 2678, Australia; ljiang@csu.edu.au (L.J.); m.dumlao@adfa.edu.au (M.C.D.); csteel@csu.edu.au (C.C.S.); 2 The Australian Research Council Training Centre for Innovative Wine Production, University of Adelaide (Waite Campus), Urrbrae, SA 5064, Australia; 3 School of Agricultural, Environmental and Veterinary Science, Faculty of Science, Charles Sturt University, Wagga Wagga, NSW 2678, Australia; 4 School of Chemistry, Faculty of Science, University of New South Wales, Sydney, NSW 2052, Australia; w.donald@unsw.edu.au

**Keywords:** quality assessment, plant disease, rapid sampling, analysis, metal–organic frameworks, thermal desorption GC-MS, VOCs

## Abstract

Fungal infection of grape berries (*Vitis vinifera*) by *Botrytis cinerea* frequently coincides with harvest, impacting both the yield and quality of grape and wine products. A rapid and non-destructive method for identifying *B. cinerea* infection in grapes at an early stage prior to harvest is critical to manage loss. In this study, zeolitic imidazolate framework-8 (ZIF-8) crystal was applied as an absorbent material for volatile extraction from *B. cinerea* infected and healthy grapes in a vineyard, followed by thermal desorption gas chromatography–mass spectrometry. The performance of ZIF-8 in regard to absorbing and trapping the targeted volatiles was evaluated with a standard solution of compounds and with a whole bunch of grapes enclosed in a glass container to maintain standard sampling conditions. The results from the sampling methods were then correlated to *B. cinerea* infection in grapes, as measured and determined by genus-specific antigen quantification. Trace levels of targeted compounds reported as markers of grape *B. cinerea* infection were successfully detected with in-field sampling. The peak area counts for volatiles 3-octanone, 1-octen-3-one, 3-octanol, and 1-octen-3-ol extracted using ZIF-8 were significantly higher than values achieved using Tenax^®^-TA from field testing and demonstrated good correlation with *B. cinerea* infection severities determined by *B. cinerea* antigen detection.

## 1. Introduction

Grapes affected by grey mould due to being infected by the fungus *Botrytis cinerea* are widespread in vineyards during certain growing periods, especially during prolonged wet and humid conditions associated with excessive rainfall or irrigation. Outbreaks of infections often occur close to grape harvest, resulting in a negative impact on yield and grape quality [1,2]. Growth of the fungus leads to the oxidisation of phenolic compounds, causing a loss of colour and the formation of a suite of off-flavours and other odours, which may impact the quality and composition of the wine and, ultimately, consumer acceptance. Monitoring fungal infection levels is essential for grape quality assessment before harvest. However, fungal infections are often hidden within the interior of the grape bunch, making visual inspections challenging. Image-based monitoring techniques may be useful but are also of limited value since they fail to detect *B. cinerea* infections hidden in the interior of the grape bunches [3]. A rapid and objective method that is suitable for assessing crop phytosanitary conditions would assist growers and winemakers as a decision support tool to decide when, or even if, to harvest. A rapid sampling and analytical method that can also be applied to monitor susceptible horticultural crops at post-harvest storage and transport stages may also assist in quality assurance of the supply chain.

Volatile metabolomes associated with *B. cinerea* infections have recently been used to establish the severity level of infection in wine grapes [4,5]. Volatile compounds elicited during fungal growth vary according to abiotic conditions, including nutrient availability, temperature, microbial interactions, and substrate [6,7]. Several volatile compounds have been selected as markers for *B. cinerea*, including 1-octen-3-ol, 1-octen-3-one, and 3-octanone, which are widely reported to cause mouldy or mushroom odours associated with fungal infections [8] and are therefore ideal targets for pathogen detection. 

The sensitive detection and quantification of hundreds of volatile compounds in complex mixtures using mass spectrometry (MS) is possible without sample pre-labelling. It is an excellent analytical approach for studying fungal biomarkers and wine off-flavours. The current MS analysis for grape composition frequently uses solid-phase microextraction (SPME) [9,10] to pre-concentrate the volatile compounds directly from the sample matrix prior to analysis by gas chromatography (GC) MS [11]. Compared with distillation or solvent extraction techniques [12], the SPME method is more time efficient and can be easily automated, and the limits of detection are usually improved [13]. Nowadays, SPME is now widely used for environmental monitoring [14,15], food and agricultural products analysis [16,17], and drug development [18].

However, due to their high cost, fragility, and relatively small volume of solid-phase materials, SPME fibres are more suitable for lab-based experiments than field sampling. To achieve in vivo sampling and real-time monitoring of volatile compounds in grapes, alternative absorbent materials encased in robust housing that is ideal for field use are desirable. Metal–organic frameworks (MOFs) have gained considerable interest due to their enormous internal surface area (>6000 m^2^/g), ultrahigh porosity (up to 90% free volume), and tuneable pore size [19] and were subsequently used for a range of analytical methods and novel applications. The unique characteristics of MOFs were used to adsorb toxic components from contaminated environments for remediation [20,21] and to lower detection limits for rapid and direct mass spectrometry analyses [22]. MOF-5 [23] was packed in quartz tubes for the in-field collection and quantification of formaldehyde, and this procedure has shown excellent stability, adsorption efficiency, and reproducibility [24]. Mixtures of aldehydes were also detected and quantified using different structured MOFs coupled with thermal desorption GC-MS and compared with a commercial absorbent, Tenax^®^-TA [25]. These applications demonstrate the range of potential usage of MOFs extraction for GC-MS in wine characterisation.

In-field static volatile sampling from plant material was performed using SPME fibres for monitoring volatiles from single grape berries and bunches of grapes in which the sampling environment was protected using glass enclosures or polyvinyl fluoride plastic bags for the duration of sampling [26]. Enclosed environments used with SPME sampling have also been created using inverted glass funnels placed over flowers to enable the monitoring of volatile emission during flower development. Static headspace sampling is an easy and non-destructive volatile collection method that is suitable for in situ fingerprint profiling and quantitative analysis of VOCs. However, the required sampling time with enclosed samples is typically very long, up to 1 day each, which substantially increases the confounding impacts of airspace humidity and heat accumulation upon volatile expression from the sample and absorption efficiency. Rapid volatile sampling methods are appropriate for high-throughput sample analysis. The reduction of sampling time can be achieved using low vacuum devices to draw air through a sampling tube packed with solid-phase material, and such devices have been deployed for a volatile sampling of Meyer lemon citrus trees and plant VOCs, enabling the detection of α–pinene, limonene, linalool and α–terpineol with as sampling duration as short as 5 min [27]. In another study, a commercially available SPME syringe coupled with an air-sampling pump was used to collect volatiles from lavender plants, *Lavandula angustifolia* and *Lavandula × intermedia*, thereby enabling species discrimination followed by a portable GC-MS for in-field volatile analysis with a 5-min sampling time and 3-min chromatographic separation [28]. An advantage of air-sampling pumps is that they produce a constant low flow rate at around 50 mL/min, allowing enough time for absorption materials to trap volatile compounds. These dynamic, volatile sampling methods allow for rapid sample extraction and analysis, demonstrating potential application for in-field or point-of-sample volatile monitoring for fruit quality assessment.

The aim of the current work was to develop a rapid, volatile sampling method for wine grapes that could be used prior to harvest to detect and measure *B. cinerea* infection. Sampling tubes with zeolitic imidazolate framework-8 (ZIF-8) crystal were applied for volatile in-field sampling and then analysed using thermal desorption GC-MS. Using a low-flow rate pump, volatile metabolites of interest were collected in 15 min for each sample, and the commercially available material Tenax^®^-TA was used for extraction, simultaneously, as a comparison. 

## 2. Results and Discussion

### 2.1. Controlled Environmental Condition for Gas Sampling

**Figure 1:**
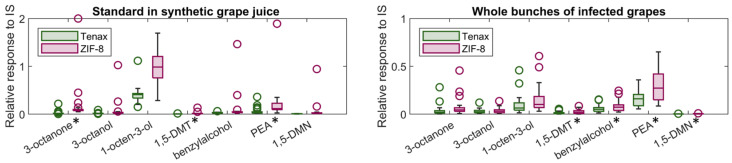
Relative responses to internal standards of some key compounds detected from synthetic grape juice with spiked standard and whole bunches of infected grapes, using ZIF-8 (red) and Tenax^®^-TA (green) as absorbent materials, detected using thermal detection GC-MS. The median marks the mid-point of the data; the middle ‘box’ represents the middle 50% of scores; the upper and lower whiskers represent scores outside the middle 50%; and the circles represent outliers of 1.5 × the interquartile range. 1,5-DMT, 1,5-dimethyltetralin; 1,5-DMN, 1,5-dimethylnaphethalene; PEA, phenylethyl alcohol; * significantly different (*p* < 0.05).

Key volatile compounds related to *B. cinerea* infection in grapes, including 3-octanone, 3-octanol, 1-octen-3-ol, 1,5-diemthyltetralin, benzyl alcohol, phenylethyl alcohol, and 1,5-dimethylnaphthalene, were detected from a controlled environment, using ZIF-8 and Tenax^®^-TA (Figure 1). In this experiment, 2-methyl-4-pentanol was applied on a filter paper as an internal standard (IS) to enable consistent volatilisation and avoid interference from grape contamination and potential absorption into the waxy berry layer, thereby confounding the analysis. The controlled environment ensured relatively stable conditions of temperature and humidity and limited air movement, so the partitioning of volatile from solution to the air within the chamber was consistent throughout the experiment. The relative response rate for most compounds using ZIF-8 was compatible with Tenax^®^-TA.

The whole bunches of *B. cinerea* control (LFD signal intensity 12–14) or infected (LFD signal intensity 37–97) grapes were placed into the same glass container system for the headspace volatile analysis, and the results were shown in Figure 1 (right). The variance for some compounds, such as phenylethyl alcohol, may be caused by the biological diversity of grapes with *B. cinerea* infection of different severities.

### 2.2. Correlation between Volatile Detection and B. cinerea Infection Severities

**Figure 2:**
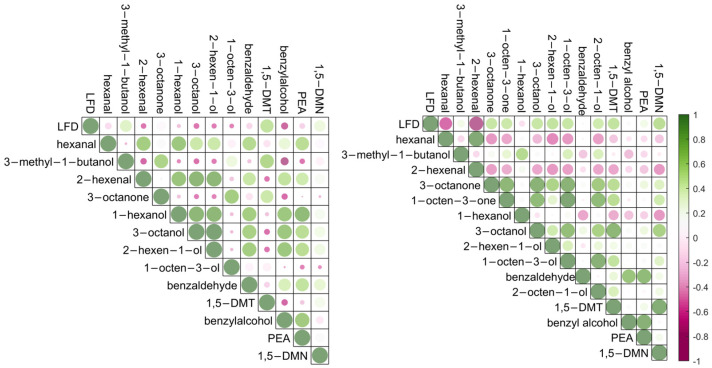
Correlation coefficients for *B. cinerea* infection levels measured with LFD scores (12–97) and volatile concentrations detected by thermal desorption GC-MS with ZIF-8 as absorbent materials, using an enclosed glass container system for volatile sampling (left), and SPME GC-MS (right). Green shows positive correlation, red shows negative correlation, and the dot sizes represent the absolute values of correlation coefficients. LFD, lateral flow device; 1,5-DMT, 1,5-dimethyltetralin; PEA, phenylethyl alcohol; 1,5-DMN, 1,5-dimethylnaphthalene.

The correlations between the *B. cinerea* infection levels and the targeted compounds detected using thermal desorption GC-MS with ZIF-8 as absorbent materials in a controlled environmental condition are shown in Figure 2 (left). *B. cinerea* infection severities were determined by the concentration of species-specific antigens, using a lateral flow device (LFD). Lateral flow devices are reported to have an excellent performance for the detection and quantification of infection levels in naturally infected grapes compared with other measures of infection, such as measurement of ergosterol [5].

In this experiment, positive correlation coefficients were observed between the LFD scores and the concentrations of 3-methyl-1-butanol, 1,5-dimethyltetralin, and 1,5-dimethylnaphthalene. 1,5-dimethyltetralin and 1,5-dimethylnaphthalene are reported as key markers for *B. cinerea* infection in grapes [5] and are thought to arise from the breakdown of berry carotenoids [29]. 3-Methyl-1-butanol was reported as a key volatile organic compound for the discrimination of bunch rot and noble rot detected in the progression of grey mould disease in grape berries [30]. In some cases, noble rot caused by *B. cinerea* is desirable, as the growth of fungi may weaken the grape skin, accelerate water evaporation, and lead to high concentrations of sugars and acids, especially for dessert wines. However, with high-humidity environments, the growth of grey mould results in the reduction of sugar accumulation and the increase of acetic acid in the juice of the grape berries which may significantly impact the wine quality. Thus, the detection of 3-methyl-1-butanol is important for harvest decisions, and volatile analysis on berries before sample crushing using a non-invasive sampling technique directly from the gas space around intact bunches provides a potential pathway for more rapid and thorough analytical support tools. Significant correlations were observed between the LFD scores and 1,5-dimethylnaphthalene, 1,5-dimethyltetralin, and 3-methyl-1-butanol, with the *p*-values of 0.011, <0.001, and 0.004, respectively (Supplementary Table S1). These metabolites are indicators of *B. cinerea* infection in grapes and strongly correlated with the infection severity. Significant differences (Supplementary Table S2) were also observed in the detection of hexanal, 3-methyl-1-butanol, 3-octanone, 1-hexanol, 3-octanol, 1,5-dimethyltetralin, benzyl alcohol, phenylethyl alcohol, and 1,5-dimethylnahphalene between healthy (LFD signal intensity 12–14) and infected bunches (LFD signal intensity 37–97), demonstrating the potential application for *B. cinerea* infection discrimination in grapes using such an enclosure glass sampling system with ZIF-8 as an absorbent material.

Apart from the correlations between the grape *B. cinerea* infection levels and the detection of the volatile compounds, Figure 2 (left) demonstrates correlations among the metabolites. For example, the concentration of 3-octanone was positively correlated with the concentrations of 3-methyl-1-butanol, 1-octen-3-ol, and 1,5-dimethyltetralin. Eight-carbon oxylipin compounds such as 3-octanone and 1-octen-3-ol are common compounds related to fungal infection in grapes possessing a typical mushroom or earthy odours [31,32,33]. They are significant metabolites produced predominantly by fungi through the enzymatic oxidation and cleavage of linoleic acid by lipoxygenase and hydroperoxide [30] and could be considered as markers for fungal infection on grapes or other horticultural crops.

SPME GC-MS was used for the analysis of the grape homogenates from the same sample set as a comparison and a benchmark method. Figure 2 (right) demonstrates the correlations between the LFD scores and the volatile metabolites detected using SPME GC-MS with the *p*-values for cross-correlation shown in Supplementary Table S3. Volatile metabolites, including 3-octanone, 3-octanol, 1-octen-3-ol, and 1-octen-3-one, were detected from *B. cinerea* infected grapes, which supports the positive correlation by thermal desorption GC-MS for the detection of 3-octanone and 1-octen-3-ol. C6 compounds hexanal and 2-henenal were negatively correlated with the *B. cinerea* grape infections and are important volatile components in grape skins derived from unsaturated fatty acids via the lipoxygenase hydroperoxide lyase enzymatic pathway, which is usually activated immediately following wounding [34,35]. Different performance of headspace volatile sampling using SPME fibre and ZIF-8 may depend on the physical or chemical properties or the detection range of the absorbent materials.

### 2.3. In-Field Volatile Sampling 

Thermal desorption GC-MS was applied for the in-field volatile sampling using ZIF-8 as absorption materials, and a commercially available material Tenax^®^-TA was used simultaneously for a comparison. In this experiment, the absolute abundances of peak area counts were integrated to compare the extraction efficiency of the two materials due to the restriction of the IS application in the field environment. This sampling experiment was conducted at a local commercial vineyard in an open-air condition with variant temperatures, humidities, and random air movement, compared to the lab-based volatile extraction experiment within a glass container. The purpose of this experiment is to demonstrate the extraction ability of fugal-related metabolites using ZIF-8 and the proof of concept for the correlations between the in-field volatile detection and the *B. cinerea* infection severities in the grapes.

**Figure 3:**
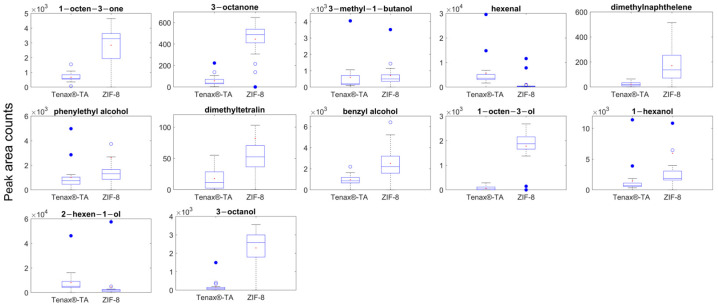
In-field sampling results of volatiles detected from *B. cinerea* infected *Chardonnay* and *Riesling*, using ZIF-8 and Tenax^®^-TA as sorbent materials with thermal desorption GC-MS. The box represents lower and upper quartile; median represents the mid-point of the data; the red dot represents the mean; the upper and lower whiskers represent scores outside the middle 50%; the open circles represent outliers of 1.5 × interquartile range (IQR) and the closed circles represent extreme outliers greater than 3 × IQR.

Volatiles related to fungal infection, hexenal, 1-octen-3-ol, 3-methyl-1-butanol, benzaldehyde, 3-octenone, *trans*-2-hexen-1-ol, 1-octen-1-one, 1-hexanol, 3-octanol, 1,5-dimethylnaphthalene, 1-5-dimethyltetralin, benzyl alcohol, and phenylethyl alcohol were readily detected using ZIF-8 and Tenax^®^-TA as absorbent materials (Figure 3). A range of volatile concentrations are evident for the in-field sample results, which reflect the range of *B. cinerea* infection severities encountered. Generally, ZIF-8 performed better with higher peak area counts for alcohol compounds, 1-octen-3-ol, 3-octanol, benzyl alcohol, and phenyl alcohol. With the formula of zinc(2-methylimidazolate)_2_, ZIF-8 is highly hydrophobic and repels adsorption of water vapour before the onset of capillary condensation, and such material was reported to be suitable for use in humid environments [36]. The selective properties of ZIF8 hydrophobic channels playing a significant role for improved affinity for bio-alcohols was demonstrated with analysis of isobutanol [37]. Our results demonstrating increased sensitivity of ZIF-8 compared to Tenax^®^-TA for the absorption of low-molecular-weight alcohols, including 1-octen-3-ol, 1-hexanol, 3-octanol, benzyl alcohol, and phenylethyl alcohol (Figure 3), are supported by prior reports of ZIF-8 selectivity. An exceptionally high capacity of ZIF8 for some alcohols is reported, which is thought to arise from the flexible structural properties of the solid-phase material, thereby increasing selectivity for this chemical class [37]. Another observation is the higher sensitivities of C8 compounds using ZIF-8 as absorbent materials, which are crucial markers for fungal infection. In this experiment, the overall peak areas detected using ZIF-8 were 8.29, 4.65, 13.13, and 18.52 times higher than Tenax^®^-TA for the detection of 3-octenone, 1-octen-3-one, 3-octanol, and 1-octen-3-ol, respectively.

The good performance of ZIF-8 for the extraction of *B. cinerea* related volatiles from in-field samples, including 3-methyl-1-butanol, 3-octanone, 1-octen-3-one, 3-octanol, 1-octen-3-ol, and phenylethyl alcohol, demonstrates the ability to apply this sorbent material to the in situ volatile collection and detection of *B. cinerea* infection in grapes prior to harvest. This non-destructive sampling method can also apply to other agricultural products and horticultural products that may be subject to fungal infection during prolonged storage or transport periods.

**Figure 4:**
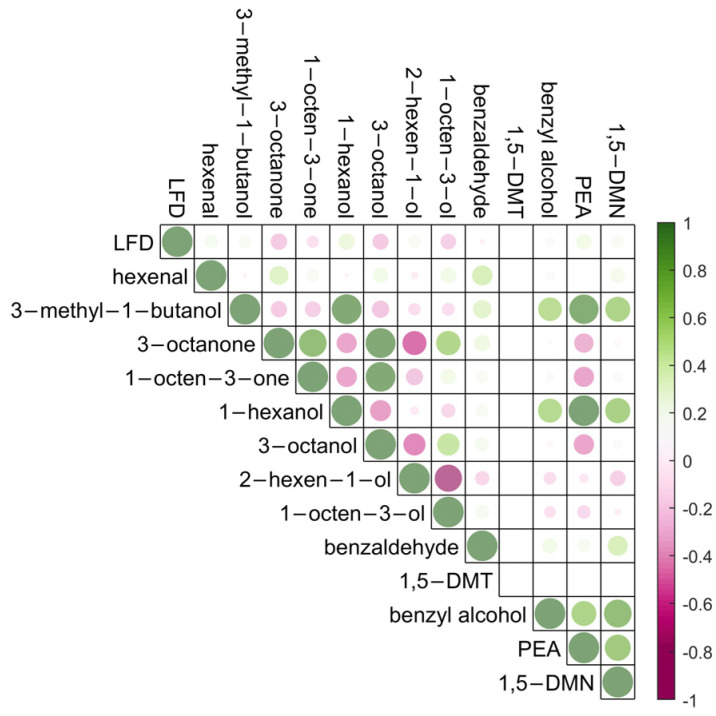
Correlation coefficient for *B. cinerea* infection levels measured with LFD scores (12–97) and the peak area counts of volatile metabolites detected by thermal desorption GC-MS, using ZIF-8 as absorbent materials, using low-flow-rate in-field sampling. Green shows positive correlation, red shows negative correlation, and the dot sizes represent the absolute values of correlation coefficients. LFD, lateral flow device; 1,5-DMT, 1,5-dimethyltetralin; PEA, phenylethyl alcohol; 1,5-DMN, 1,5-dimethylnaphthalene.

A correlation coefficient plot to show the relationship between the grape *B. cinerea* infection and the detection of volatile compounds is presented in Figure 4. Strong positive correlations were observed between the detection of 1-octen-3-one, 3-octanone, 3-octanol, and 1-octen-3-ol, as well as the detection of 3-methyl-1-butanol, 1-hexanol, 1,5-dimethyltetralin, benzyl alcohol, phenylethyl alcohol, and 1,5-dimethylnaphthalene for in-field results. These volatiles were important for *B. cinerea* grape infection, and the correlations may be explained by the growth progression of the pathogen. For example, 3-methyl-1-butanol and phenylethyl alcohol were reported as key volatiles for noble rot and grey mould discrimination [30]; and 3-octanol, phenylethyl alcohol, and 1,5-dimethylnaphthalene were regarded as key biomarkers for the detection and prediction of *B. cinerea* infection in grapes [5]. The detection of hexenal and 3-octanone was significantly different (Supplementary Table S2) between healthy (LFD signal intensity 12–14) and infected bunches (LFD signal intensity, 37–97), with *p*-values of 0.080 and 0.034, respectively.

Compared to the sampling in controlled conditions (Figure 2, left), relatively weak correlations were observed between the LFD scores and the detection of 3-methyl-1-butanol, 1,5-dimethyltetralin, phenylethyl alcohol, and 1,5-dimethylnaphthalene from in-field sampling (Supplementary Table S4). The apparent difference between sampling strategies is likely to be related to the uncontrolled environmental conditions encountered during the in-field sampling of grape bunches. Whilst every effort was made to position extraction tubes closely and evenly to grape bunches, changing conditions during the day will alter the relative volatility of the target molecules. Variances in sample-extraction efficacy with controlled environments are corrected through the use of the IS [38]; however, their incorporation into a suite of target compounds is not possible in the field. 

Key volatile metabolites were able to be extracted from in-field sampling using ZIF-8 as absorbent materials, thus demonstrating a proof of concept for in-field sampling for the detection of *Botrytis cinerea* infection. The use of a hermetically sealed controlled environment such as a glass container to eliminate air movement and maintain standard sampling conditions enabled a higher level of sensitivity for some volatiles to be achieved for ZIF8 compared to Tenax^®^-TA.

## 3. Materials and Methods

### 3.1. Chemical and Materials

Analytical-grade chemicals and reagents for preparing standard solutions for GC-MS analysis were used without further purification. Standard 1-octen-3-one (96%), 1-octen-3-ol (≥98%), 3-octanone (≥98%), benzaldehyde (≥99.5%), *trans*-2-hexen-1-ol (96%), benzyl alcohol (99%+), 1,5-dimethyl naphthalene (98%), 3-methyl-1-butanol (99%+), 1,5-dimethyltetralin (≥90%), phenylethyl alcohol (≥99%), 4-methyl-2-pentanol (98%), glucose (≥99.5%), fructose (≥99%), tartaric acid (99%), naphthalene-d8 (99 atom% D, ≥98% (CP)), 3-octanol (99%), 2-octanol (≥99.5%), zinc nitrate hexahydrate (98%), 2-methylimidazole (98%), and glass wool were purchased from Sigma-Aldrich (Sydney, NSW, Australia). Hexanal (98%), 1-hexanol (99%), menthol (HPLC), and *N,N*-dimethylformamide (DMF) (99%) were purchased from Alfa Aesar (Scoresby, VIC, Australia). Hexanal-d12 (98.5 atom % D) and 2,4,6-tribromoanisol-d5 (99 atom % D) were purchased from CDN Isotopes (Pointe-Claire, Quebec, Canada). Milli-Q™ water (18 MΩ·cm) was collected through a Millipore™ water purification system (Merk, Bayswater, VIC, Australia). Synthetical grape juice was made using 100 g glucose, 100 g fructose, and 4 g tartaric acid. All solutions were prepared in Milli-Q™ water, with the pH adjusted to 7.0. 

### 3.2. ZIF-8 Synthesis and Sampling Tube Preparation

ZIF-8 were synthesised based on a previous reported study [39]. In brief, 0.67 g (2 mmol) of zinc nitrate hexahydrate, 0.167 g (2 mmol) of 2-methylimidazole, and 50 mL *N,N*-dimethylformamide were mixed with 5 min of sonication in a 100 mL screw-thread vial. The mixture was then heated at 140 °C for 24 h in a convection oven and then filtered and washed with DMF several times, kept in methanol for 3 days, and dried at room temperature.

The volatile sampling tube was prepared in a pretreated empty thermal desorption tube (O.D. × L 1/4 in. × 3 ½, LECO Australia Pty. Ltd., Castle Hill, NSW, Australia) with 250 mg of dried ZIF-8, making it a comparable weight to a commercially available sorbent tube, Markes Tenax^®^-TA (Kinesis Australia, Redland Bay, QLD, Australia). Each tube was packed in the following sequence: stainless-steel gauze disc, ZIF-8, glass wool, and stainless-steel G-Clip (Kinesis Australia, Redland Bay, QLD, Australia). Prior to sampling, the ZIF-8 and Tenax^®^-TA sorbent tubes were conditioned using a Markes TC-20 tube conditioner system (Kinesis Australia, Redland Bay, QLD, Australia), with a 40 mL/min nitrogen flow at 300 °C for 2 h. 

### 3.3. VOCs Extraction Using ZIF-8

#### 3.3.1. Volatile Extraction in a Closed Environment

Volatile metabolites were extracted from the headspace of whole bunches of the same grapes harvested from the in-field sampling experiment, using an enclosed glass container. Ten *Chardonnay* and ten *Riesling* grape bunches were collected from a local vineyard in Tumbarumba, NSW, Australia, and *B. cinerea* infection severities were measured using a lateral flow device (Section 3.5).

**Figure 5:**
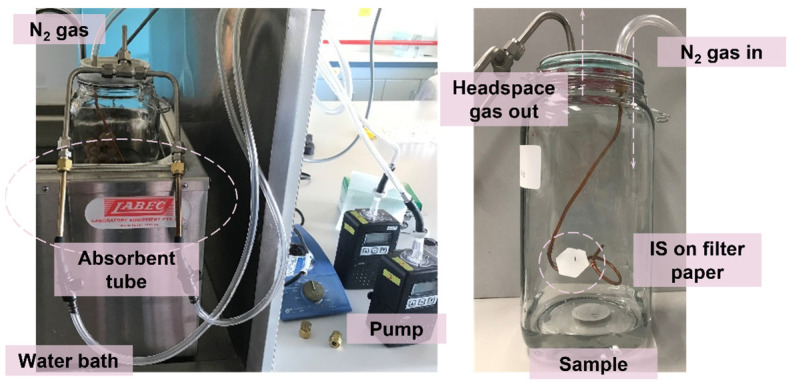
Headspace volatile extraction in a 2.5 L glass container, using ZIF-8 and Tenax^®^-TA as sorbent materials. A solution of internal standard was applied on filter paper.

Sampling for volatiles from a standard mix of target volatile compounds and from naturally infected bunches of grapes, assessed for visible signs of grey mould, was undertaken using a 2.5 L glass container to control the temperature and air movement. Synthetic grape juice (100 mL, as described previously) spiked with 50 μL mixed standard in methanol to a final concentration of 7.2 μg/L 1-octen-3-one, 93.6 7.2 μg/L 3-octanol, 32.8 μg/L 1-octen-3-ol, 2.39 μg/L 1,5-diemthyltetralin, 1690 μg/L benzyl alcohol, 247 μg/L phenylethyl alcohol, and 3.39 μg/L 1,5-dimethylnaphthalene in the container, or individual grape bunches were placed into the container prior to insertion of the IS. An IS comprising 10 μL 40 mg/L 2-methyl-4-pentanol in methanol was applied on a cut filter paper disc (200 mm, Whatman #1, Sigma-Aldrich, Sydney, NSW, Australia) fixed onto a copper wire inside the glass container (Figure 5). The paper discs were heated at 120 °C for 2 h before they were used to remove any impregnated volatiles via volatilization. Prior to sampling, the glass container was placed in a water bath for pre-heating at 30 °C for 5 min to simulate the field environment. During the headspace sampling process, a ZIF-8 tube, and a Tenax^®^-TA sorbent tube were connected in parallel for simultaneous sampling. As shown in Figure 5, a SKC pump was connected to the absorbent tube for a 15 min extraction at 100 mL/min, and nitrogen gas was applied at 200 mL/min for pressure equivalent. 

#### 3.3.2. In-Field Volatile Extraction

**Figure 6:**
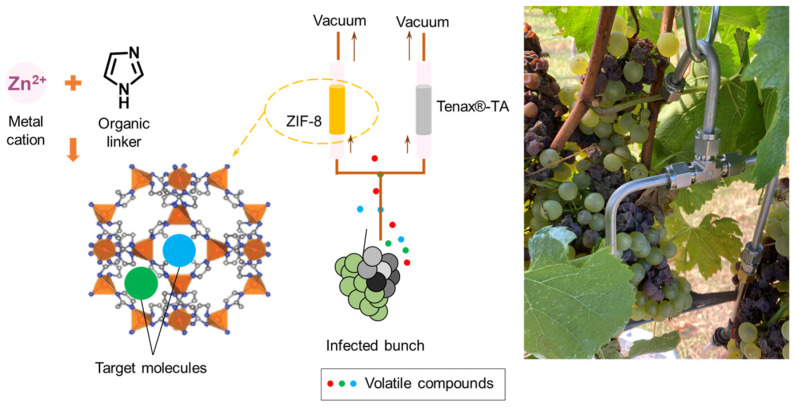
Diagram of in-field volatile sampling experiment. Gas sampling tubes with ZIF-8 and Tenax^®^-TA were connected using a ‘T’ junction and used to simultaneously extract volatile compounds from a bunch of Chardonnay grapes.

In-field sampling was performed at a local vineyard in Tumbarumba, NSW, Australia. Volatile metabolites were extracted from 10 *Chardonnay* bunches and 10 *Riesling* bunches of different *B. cinerea* infection severities. During in-field sampling, a T-shaped stainless-steel tube was used to connect a ZIF-8 tube and a Tenax^®^-TA sorbent tube, allowing the two tubes to be used simultaneously. The other side of the T-shaped tube was placed just next to a grape bunch (Figure 6). A low-flow-rate air-sampling pump (AirCheck XR5000 Pump, SKC Ltd., Blandford, UK) was connected to each tube to create an air flow of 100 mL/min for 15 min of dynamic extraction. The whole-grape bunches for this experiment were harvested and stored at −20 °C until further analysis. 

### 3.4. VOCs Detection Using GC-MS 

#### 3.4.1. Thermal Desorption Procedure

Thermal desorption was processed with a Markes UNITY-xr single-tube thermal desorption unit (Kinesis Australia, Redland Bay, QLD, Australia) connected to a GC-MS system by a transfer line maintained at 150 °C with a constant nitrogen flow at 10 mL/min. The sampling tubes were pre-desorbed for 1 min with a trap flow of 50 mL/min and then thermally desorbed at 250 °C for 10 min with the same flow rate and then re-trapped at 30 °C for 1 min by a Markes focusing (cold) trap made of quartz and 60 mm graphitised carbon (General Purpose, Kinesis Australia, Redland Bay, QLD, Australia). The direction of the gas flow was always reversed during the desorption process, enabling higher boiling compounds to backflash from the sampling end of the tube. After analyte concentration, the trap was flash heated to 300 °C for 3 min at with the split flow of 20 mL/min. A moisture control system was applied to remove water vapour before sample injection to the GC-MS system. 

#### 3.4.2. SPME Extraction Parameter

Individual grape berries were carefully removed from bunches after whole-bunch volatile extraction and homogenised using a high-speed grinder (UltraTurrax T25 IKA Dispersers, Staufen, Germany). The grape homogenates were stored at −20 °C until immediately prior to analysis. During SPME analysis, 2.5 mL of grape homogenate was mixed with 7.5 mL Milli-Q water, 3 g sodium chloride, and 10 μL of the IS solution, as previously reported [5] in a 20 mL headspace glass vial (Agilent Technologies Ltd., Mulgrave, VIC, Australia). All samples were analysed in randomised order, and the vials were vortexed for 10 s before analysis. 

During SPME extraction, a DVB/CAR/PDMS fibre (Stableflex 23 Ga fibre; Sigma-Aldrich, Sydney, NSW, Australia) was used with an MPS2XL Multipurpose Sampler (Gerstel Inc., Linthicum, MD, USA), a Peltier sample cooler tray, and a heated agitator stirrer. Sample vials were placed into the Peltier sample cooler (4 °C) before analysis. Vials were subsequently transferred into the heated oven (65 °C), where they were incubated for 2 min before insertion of the SPME fibre for 64 min under agitation at 250 rpm. Injection of the SPME fibre into an ultra-inert straight SPME liner (0.75 mm; Agilent Technologies Ltd., Mulgrave, VIC, Australia) was performed at an inlet temperature of 260 °C in split-less mode for 1 min. The fibre was then baked for 10 min at 270 °C in a secondary injector. 

The calibration curves for SPME GC-MS were established using mixed standard solutions prepared in synthetic grape juice at six concentrations, in triplicate. The calibration curves and specific GC parameters were previously reported [5]. A mix of internal standards was added to each sample/standard vial to achieve the final concentration of hexanal-d12 (6.25 μg/L), 2-octanol (0.22 μg/L), naphehalene-d8 (0.10 μg/L), 2,4,6-trichloroanisole-d5 (0.15 μg/L), and phenol-d6 (3.00 μg/L).

#### 3.4.3. GC-MS Parameters and Statistics Processing

An Agilent 7890A gas chromatography coupled with an Agilent 5975C triple-axis mass selective detector (Agilent Technologies Ltd., Mulgrave, VIC, Australia) was used for MS detection with a DB-Waxetr column (60 m × 250 μm inner diameter × 0.25 μm film thickness; Agilent Technologies Ltd., Mulgrave, VIC, Australia). The GC method was based on a published study for volatiles detection from fungal-infected grapes [9]. In brief, the helium gas flow rate was held at 3 °C/min, with an initial oven temperature of 40 °C, held for 3 min before heating at 5 °C/min until 115 °C; increased at 2 °C/min until 166 °C; and then increased at 8 °C/min until 240 °C was reached. The final temperature was held for 15 min, resulting in a total run time of 67.75 min. Source, quadrupole, and transfer line temperatures were set to 230 °C, 150 °C, and 280 °C, respectively. After a solvent delay of 8 min, the mass detector recorded a range between mass ion ratios (*m*/*z*) of 35–550, using selective ion monitoring/scan mode, according to a previously reported study [5], at −70 eV. The mass spectral data were acquired using the Enhanced MSD ChemStation software (version E.02.00.493, Agilent Technologies Ltd., Mulgrave, VIC, Australia) and assessed using NIST MS search (version 2.0).

Raw data MS data were analysed using Agilent MassHunter Quantitative Analysis (MS, version 8.0, Agilent Technologies Ltd., Mulgrave, VIC, Australia). The limit of detection (LOD) and limit of quantification (LOQ) were calculated with concentrations for signal-to-noise ratios of three and ten, respectively. Peak areas, relative responses, and calculated concentrations of each analyte detected from grape samples were imported into MATLAB (version R2022a, The MathWorks Inc., Novi, MI, USA). 

### 3.5. Botrytis cinerea Quantification

*B. cinerea* infection severities were determined by *B. cinerea* antigen measurement using a lateral flow device (LFD; Global Access Diagnostics (GADX), Thurleigh, Bedfordshire, UK) with the *B. cinerea* monoclonal antibody, BC-12.CA4, as the target, as previously reported [5]. In brief, 2 mL of grape homogenates were centrifuged at 3000× *g* for 10 min, followed by 1/1000 dilution with a 0.1% (*v/v*) Tween 20 solution in phosphate-buffered saline (PBS; Sigma-Aldrich, Sydney, NSW, Australia). After that, a 200 µL aliquot of the diluted homogenates was applied into a 2 mL Eppendorf tubes at room temperature. A *B. cinerea* test strip (GADX, Thurleigh, Bedfordshire, UK) was placed into each tube and read using a cube reader (GADX, Thurleigh, Bedfordshire, UK) after 10 min. The signal intensities were recorded as LFD scores described in the manufacturer’s instructions.

## 4. Conclusions

In this experiment, volatile metabolites related to *B. cinerea* grape infection were detected using ZIF-8 as absorbent materials for thermal desorption GC-MS during in-field volatile sampling experiments. When used with stable environmental conditions incorporating an internal standard to improve sampling efficacy, the ZIF8 absorbent resulted in showing excellent correlations between the target volatile compounds’ *B. cinerea* grape infection antigen LFD scores. The performance of key markers for fungal infection, 3-octanone, 3-ocatnol, 1-octen-3-one, and 1-octen-ol, using ZIF-8, demonstrated improved sensitivity when compared to the commercially available material, Tenax^®^-TA. Positive correlations between the detection of key *B. cinerea* grape infection markers, such as 1,5-dimethyltetralin and the LFD scores, were observed, indicating that this sampling collection method may be potentially applied for the detection and prediction of grape *B. cinerea* infection in field. This series of experiments demonstrated a proof of concept for the use of tuneable and selective absorbent solid-phase materials for the in-filed sampling of targeted compounds.

## Data Availability

The raw data are available from the corresponding author upon reasonable request.

## Supplementary Materials

The following supporting information can be downloaded at https://www.mdpi.com/article/10.3390/molecules28135227/s1, Table S1: The *p*-values for cross-correlation between LFD signal intensity representing *B. cinerea* infection severities and volatile metabolites detection in grapes by thermal desorption GC-MS with ZIF-8 as absorbent materials, using an enclosed glass container system for sampling. LFD, lateral flow device; 1,5-DMT, 1,5-dimethyltetralin; PEA, phenylethyl alcohol; 1,5-DMN, 1,5-dimethylnaphthalene, Table S2: The *p*-values for healthy (LFD = 12–14) and *B. cinerea* infected (LFD = 37–97) grape bunches. LFD, lateral flow device; 1,5-DMT, 1,5-dimethyltetralin; PEA, phenylethyl alcohol; 1,5-DMN, 1,5-dimethylnaphthalene, Table S3: The *p*-values for cross-correlation between LFD signal intensity representing *B. cinerea* infection severities and volatile metabolites detection in grapes by SPME GC-MS. LFD, lateral flow device; 1,5-DMT, 1,5-dimethyltetralin; PEA, phenylethyl alcohol; 1,5-DMN, 1,5-dimethylnaphthalene, Table S4: The *p*-values for cross-correlation between LFD signal intensity representing *B. cinerea* infection severities and volatile metabolites detection in grapes by thermal desorption GC-MS with ZIF-8 as absorbent materials, using low-flow-rate in-field sampling. LFD, lateral flow device; 1,5-DMT, 1,5-dimethyltetralin; PEA, phenylethyl alcohol; 1,5-DMN, 1,5-dimethylnaphthalene.

## Sample Availability

Samples of the compounds are not available from the authors.
