# Supplementary material for: Rapid In-Field Volatile Sampling for Detection of Botrytis cinerea Infection in Wine Grapes
Source: Molecules. 2023 Jul 5;28(13):5227. doi: 10.3390/molecules28135227 (PMC10343791; doi:10.3390/molecules28135227)
Supplement: Supplementary file 1 [file molecules-28-05227-s001.zip › molecules-2453240-supplementary.pdf]

Rapid In-Field Volatile Sampling for Detection of *Botrytis cinerea* Infection in Wine Grapes

Supplementary Data

Liang Jiang [1-3], Morphy C. Dumlao [1,2,4], William A. Donald [4], Christopher C. Steel [1,3], and Leigh M. Schmidtke [1-3,*]

1 Gulbali Institute, Charles Sturt University, Wagga Wagga, NSW, Australia 2678

2 The Australian Research Council Training Centre for Innovative Wine Production, University of Adelaide (Waite Campus), SA, Australia 5064

3 School of Agricultural, Environmental and Veterinary Science, Faculty of Science, Charles Sturt University, Wagga Wagga, NSW, Australia 2678

4 School of Chemistry, Faculty of Science, University of New South Wales, NSW, Australia 2052

* Correspondence: lschmidtke@csu.edu.au; Tel.: +61 2 6933 2016

Table S1. *p*-values for cross correlation between LFD signal intensity representing *B. cinerea* infection severities and volatile metabolites detection in grapes by thermal desorption GC-MS with ZIF-8 as absorbent materials using an enclosed glass container system for sampling. LFD: lateral flow device; 1,5-DMT: 1,5-dimethyltetralin; PEA: phenylethyl alcohol; 1,5-DMN: 1,5-dimethylnaphthalene.

	LFD	hexanal	3-methyl-1-butanol	2-hexenal	3-octanone	1-hexanol	3-octanol	2-hexen-1-ol	1-octen-3-ol	benzaldehyde	1,5-DMT	BENZYL ALCOHOL	PEA	1,5-DMN
1,5-DMN	0.011	0.093	0.185	0.036	0.89	0.029	0.02	0.015	0.781	0.007	0.026	0.246	0.073	1
PEA	0.536	<0.001	0.584	0.001	0.968	<0.001	<0.001	<0.001	0.677	<0.001	0.704	<0.001	1	
benzyl alcohol	0.356	0.008	0.23	<0.001	0.48	<0.001	<0.001	<0.001	0.937	0.002	0.45	1		
1,5-DMT	<0.001	0.324	<0.001	0.568	0.001	0.753	0.527	0.562	0.165	0.532	1			
benzaldehyde	0.482	<0.001	0.749	<0.001	0.34	<0.001	<0.001	<0.001	0.152	1				
1-octen-3-ol	0.686	0.184	0.012	0.701	<0.001	0.793	0.846	0.845	1					
2-hexen-1-ol	0.625	0.001	0.613	<0.001	0.659	<0.001	0	1						
3-octanol	0.549	0.001	0.547	<0.001	0.639	<0.001	1							
1-hexanol	0.762	<0.001	0.745	<0.001	0.773	1								
3-octanone	0.087	0.084	<0.001	0.991	1									
2-hexenal	0.573	<0.001	0.449	1										
3-methyl-1-butanol	0.004	0.897	1											
PEA	0.404	1												
LFD	1													

Table S2. *p*-values for healthy (LFD = 12-14) and *B. cinerea* infected (LFD = 37-97) grape bunches. LFD: lateral flow device; 1,5-DMT: 1,5-dimethyltetralin; PEA: phenylethyl alcohol; 1,5-DMN: 1,5-dimethylnaphthalene.

	hexanal	*3-methyl-1-butanol*	*3-octanone*	*1-hexanol*	*3-octanol*	*2-hexen-1-ol*	*1-octen-3-ol*	*benzaldehyde*	*1,5-DMT*	*benzyl alcohol*	*PEA*	*1,5-DMN*
Enclosure sampling in glass system	<0.001	0.008	<0.001	0.046	<0.001	0.072	0.149	0.07	<0.001	0.005	0.041	<0.001
SPME GC-MS	0.054	<0.001	<0.001	0.235	<0.001	<0.001	<0.001	0.499	0.001	0.428	0.023	<0.001
In-field sampling	0.08	0.374	0.034	0.142	0.136	0.138	0.421	0.346	0.189	0.105	0.28	0.462

Table S3. *p*-values for cross correlation between LFD signal intensity representing *B. cinerea* infection severities and volatile metabolites detection in grapes by SPME GC-MS. LFD: lateral flow device; 1,5-DMT: 1,5-dimethyltetralin; PEA: phenylethyl alcohol; 1,5-DMN: 1,5-dimethylnaphthalene.

	LFD	*hexanal*	*3-methyl-1-butanol*	*2-hexenal*	*3-octanone*	*1-octen-3-one*	*1-hexanol*	*3-octanol*	*2-hexen-1-ol*	*1-octen-3-ol*	*benzaldehyde*	*2-octen-1-ol*	*1,5-DMT*	*benzyl alcohol*	*PEA*	*1,5-DMN*
1,5-DMN	0.003	0.13	0.736	0.017	0.046	0.02	0.016	<0.001	0.557	0.057	0.256	0.18	<0.001	0.776	0.28	1
PEA	0.228	0.393	0.34	0.14	0.163	0.775	0.222	0.168	0.439	0.591	<0.001	0.818	0.222	<0.001	1	
benzyl alcohol	0.698	0.58	0.1	0.332	0.463	0.809	0.103	0.506	0.417	0.979	<0.001	0.981	0.639	1		
1,5-DMT	0.006	0.157	0.794	0.035	0.002	0.002	0.044	<0.001	0.223	0.008	0.14	0.033	1			
2-octen-1-ol	0.019	0.019	0.04	0.029	<0.001	<0.001	0.664	<0.001	0.13	<0.001	0.907	1				
benzaldehyde	0.934	0.946	0.146	0.49	0.526	0.892	0.033	0.21	0.633	0.938	1					
1-octen-3-ol	0.01	0.015	0.042	0.021	<0.001	<0.001	0.391	<0.001	0.033	1						
2-hexen-1-ol	0.007	0.007	0.835	0.013	<0.001	0.075	0.936	0.02	1							
3-octanol	0.009	0.071	0.59	0.032	<0.001	<0.001	0.577	1								
1-hexanol	0.474	0.634	0.002	0.335	0.645	0.566	1									
1-octen-3-one	0.015	0.03	0.106	0.04	<0.001	1										
3-octanone	0.01	0.015	0.382	0.02	1											
2-hexenal	<0.001	<0.001	0.38	1												
3-methyl-1-butanol	0.25	0.342	1													
hexanal	0.001	1														
LFD	1															

Table S4. *p*-values for cross correlation between LFD signal intensity representing *B. cinerea* infection severities and volatile metabolites detection in grapes by thermal desorption GC-MS with ZIF-8 as absorbent materials using low flow rate in-field sampling. LFD: lateral flow device; 1,5-DMT: 1,5-dimethyltetralin; PEA: phenylethyl alcohol; 1,5-DMN: 1,5-dimethylnaphthalene.

	LFD	*hexanal*	*3-methyl-1-butanol*	*2-hexenal*	*3-octanone*	*1-hexanol*	*3-octanol*	*2-hexen-1-ol*	*1-octen-3-ol*	*benzaldehyde*	*1,5-DMT*	*benzyl alcohol*	*PEA*	*1,5-DMN*
1,5-DMN	0.473	0.322	0.002	0.77	0.598	0.002	0.675	0.283	0.823	0.068	<0.001	<0.001	0.001	1
PEA	0.294	0.986	<0.001	0.091	0.062	<0.001	0.058	0.697	0.424	0.42	<0.001	0.002	1	
benzyl alcohol	0.649	0.685	0.008	0.967	0.715	0.005	0.857	0.486	0.552	0.308	0.001	1		
1,5-DMT	0.291	0.764	<0.001	0.144	0.106	<0.001	0.096	0.596	0.493	0.302	1			
benzaldehyde	0.91	0.063	0.126	0.249	0.47	0.463	0.392	0.368	0.385	1				
1-octen-3-ol	0.29	0.284	0.468	0.004	0.292	0.378	0.019	<0.001	1					
2-hexen-1-ol	0.443	0.816	0.508	0.005	0.197	0.737	0.022	1						
3-octanol	0.251	0.32	0.186	<0.001	<0.001	0.042	1							
1-hexanol	0.233	0.932	<0.001	0.064	0.055	1								
3-octanone	0.51	0.476	0.263	<0.001	1									
2-hexenal	0.23	0.106	0.244	1										
3-methyl-1-butanol	0.451	0.931	1											
hexanal	0.416	1												
LFD	1													
